# Colorimetric Detection of the SARS-CoV-2 Virus (COVID-19) in Artificial Saliva Using Polydiacetylene Paper Strips

**DOI:** 10.3390/bios12100804

**Published:** 2022-09-29

**Authors:** Christopher D. Prainito, Gaddi Eshun, Francis J. Osonga, Daniel Isika, Cynthia Centeno, Omowunmi A. Sadik

**Affiliations:** 1 Harvard University, 29 Oxford Street, Cambridge, MA 02138, USA; 2 Chemistry and Environmental Science Department, New Jersey Institute of Technology, University Heights, Newark, NJ 07102, USA

**Keywords:** SARS-CoV-2, biosensors, paper strips, iPhone readout, selective and rapid detection, antibody, saliva

## Abstract

The spread and resurgence of the SARS-CoV-2 virus (COVID-19 disease) threatens human health and social relations. Prevention of COVID-19 disease partly relies on fabricating low-cost, point-of-care (POC) sensing technology that can rapidly and selectively detect the SARS-CoV-2 virus. We report a colorimetric, paper-based polydiacetylene (PDA) biosensor, designed to detect SARS-CoV-2 spike protein in artificial saliva. Analytical characterizations of the PDA sensor using NMR and FT-IR spectroscopy showed the correct structural elucidation of PCDA-NHS conjugation. The PDA sensor platform containing the N-Hydroxysuccinimide ester of 10, 12-pentacosadiynoic acid (PCDA-NHS) was divided into three experimental PCDA-NHS concentration groups of 10%, 20%, and 30% to optimize the performance of the sensor. The optimal PCDA-NHS molar concentration was determined to be 10%. The PDA sensor works by a color change from blue to red as its colorimetric output when the immobilized antibody binds to the SARS-CoV-2 spike protein in saliva samples. Our results showed that the PDA sensing platform was able to rapidly and qualitatively detect the SARS-CoV-2 spike protein within the concentration range of 1 to 100 ng/mL after four hours of incubation. Further investigation of pH and temperature showed minimal influence on the PDA sensor for the detection of COVID-19 disease. After exposure to the SARS-CoV-2 spike protein, smartphone images of the PDA sensor were used to assess the sensor output by using the red chromatic shift (RCS) of the signal response. These results indicate the potential and practical use of this PDA sensor design for the rapid, colorimetric detection of COVID-19 disease in developing countries with limited access to medical testing.

## 1. Introduction

Coronavirus disease (COVID-19) is an ongoing global pandemic with increased mutation and health complications. Severe Acute Respiratory Syndrome Coronavirus 2 (SARS-CoV-2) causes COVID-19 disease, and over 250 million cases have been confirmed [1,2,3]. Thus, adopting effective surveillance and monitoring protocols is important in controlling the resurgence of COVID-19. The *p*-Conjugated polydiacetylene sensor platforms (PDAs) are a promising choice for the non-invasive detection of COVID-19 disease. PDAs possess an intrinsic chromatic property, and the disruption of their pi-conjugated systems translates into a color change from blue to red [4]. This knowledge has been utilized to design colorimetric sensors for pathogenic viruses such as influenza [5]. Our goal is to design a low-cost, colorimetric sensing platform using a PDA-coated paper strip that can selectively detect COVID-19 disease in saliva samples.

To date, multiple detection methods such as Reverse Transcription Polymerase Chain Reaction (RT-PCR) [6], immunoassays [7], and RT-qPCR [8] have been developed to detect COVID-19 disease [9]. Some disadvantages associated with RT-qPCR include the need for trained personnel and expensive RT-qPCR reagent kits, which limit its extensive application. Furthermore, although RT-PCR analysis can process several samples simultaneously [10], samples with a viral load below the assay’s limit of detection (LOD) could result in false negatives [11,12,13]. These limitations require the fabrication of new, low-cost, and reliable point-of-care (POC) devices.

POC diagnostic technology is increasingly popular for detecting COVID-19 disease due to its low complexity, quick processing time, and portability [14]. Paper-based sensors fit the criteria for POC testing due to their flexibility and portability [15]. Thus, paper-based sensors are suitable for real-time testing and are ideal for use in developing countries with limited resources [16,17]. Furthermore, the sample acquisition of saliva is an acceptable alternative specimen type for SARS-CoV-2 detection using POC devices [18,19].

Polymer-based biosensors serve as POC technology for detecting viruses due to their low-cost and high sensitivity. Polydiacetylenes (PDAs) are a unique class of linear polymers produced by 1,4-addition polymerization of diacetylene monomers by UV irradiation [20]. PDAs are excellent candidates for designing colorimetric biosensors owing to their chromatic properties. Polydiacetylenes undergo a characteristic blue to red color change when exposed to a targeted protein [21]. This visual, color-based transition makes PDAs ideal sensors because the stimulated change can be visualized or determined by a spectrometer [5,22].

In this work, a colorimetric PDA-based paper biosensor has been developed to rapidly detect COVID-19 disease in artificial saliva. The PDA biosensor was fabricated by coating the PDA on a polyvinylidene fluoride (PVDF) paper strip through an evaporation process. A phosphate-buffered saline (PBS) solution of SARS-CoV-2 spike protein antibody interacted with three sets of PDA formulations through a PCDA-NHS reaction. The immersion of PDA-coated paper strips in PBS containing SARS-CoV-2 spike protein antibody showed a remarkable color change upon exposure to UV light. Smartphone imaging was used to determine the colorimetric shift of the sensing membrane after exposure to the SARS-CoV-2 spike protein in PBS and artificial saliva solutions. An optimal concentration of PCDA-NHS was determined to maximize the sensor’s colorimetric shift in the presence of the SARS-CoV-2 spike protein to facilitate the colorimetric response. The versatility, low-cost, and easy-to-use PDA biosensor in this study necessitates its real-life application.

## 2. Materials and Methods

### 2.1. Materials

10,12-pentacosadiynoic acid (PCDA) was purchased from Sigma-Aldrich (St. Louis, MO, USA). 1,2-dimyristoyl-sn-glycerol-3-phosphocholine (DMPC) was purchased from Avanti Polar Lipids (Alabaster, AL, USA). PVDF transfer membranes (0.45 µm) were purchased from Thermo Fisher Scientific (Waltham, MA, USA). Artificial saliva with mucin (Pickering Laboratories) was purchased from Fisher Scientific (Waltham, MA, USA). SARS-CoV-2 Spike S1 Antibody, SARS-CoV-2 Spike S1-His Recombinant Protein, and MERS-CoV Spike S1 Protein were purchased from Sino Biological (Beijing, China). All other compounds were purchased from Sigma-Aldrich (St. Louis, MO, USA).

### 2.2. Synthesis of PCDA-NHS Monomer

The synthesis of the N-Hydroxysuccinimide (NHS) ester of PCDA (PCDA-NHS) was performed as previously reported [5,23]. Briefly, 2.00 g (5.4 mmol) of PCDA, 675 mg (5.8 mmol) of NHS, and 1.23 g (6.4 mmol) of 1-Ethyl-3-(3-dimethylaminopropyl) carbodiimide (EDC) were dissolved in 20 mL of dichloromethane. The solution was then homogenized with a magnetic stirrer for 4 h at room temperature (20 °C). The solvent was evaporated to dryness using a rotary evaporator, and the residue was purified by extraction with ethyl acetate and distilled water mixture. Following separation, the organic layer was filtered using magnesium sulfate anhydrous, and the solvent was evaporated to dryness using a rotary evaporator. Finally, the residue was removed from the rotary evaporator to yield PCDA-NHS as a white powder (1.62 g). To confirm that the PCDA-NHS synthesis reaction had been completed, thin-layer chromatography (TLC) was performed using hexane and ethyl acetate (3:1) as the solvent system to monitor the reaction products. PCDA, NHS, and PCDA-NHS powders were then dissolved in ethyl acetate, spotted onto a silica plate, and placed in the developing chamber.

### 2.3. Characterization of PCDA and PCDA-NHS

The chemical structure of PCDA and synthesis of PCDA-NHS were characterized using both Fourier-Transform Infrared spectroscopy (FT-IR) and Proton Nuclear Magnetic Resonance (^1^H-NMR; 500 MHz, DMSO-d_6_).

### 2.4. Preparation of Polydiacetylene Sensing Chips

**Figure 1:**
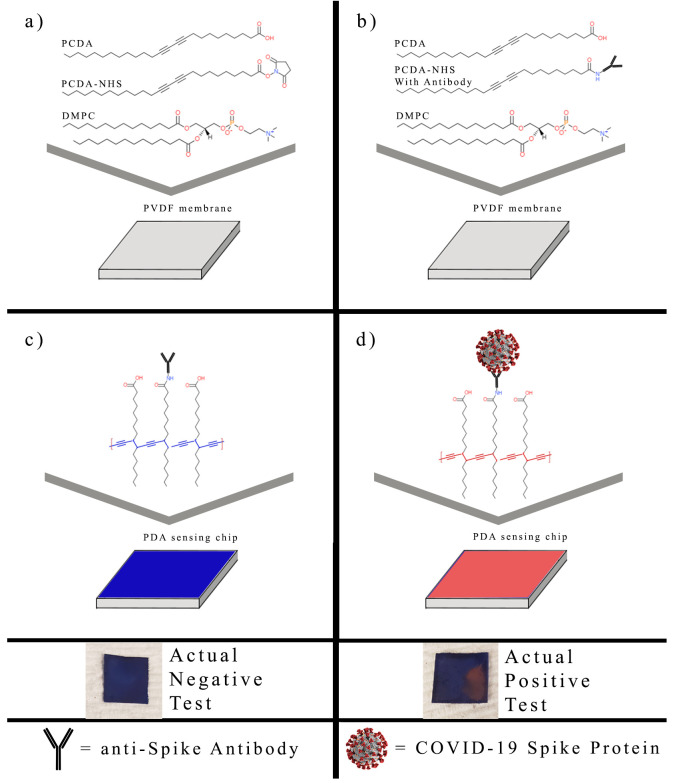
Creation of polydiacetylene-based sensor. PVDF membranes were dipped into the prepared chloroform solutions (**a**), incubated in solutions containing the SARS-CoV-2 spike protein antibody (**b**), photopolymerized by 254 nm UV light (**c**), and exposed to the SARS-CoV-2 spike protein in artificial saliva and PBS buffer (**d**). Images are shown of PCDA-NHS-1 sensors exposed to unmodified artificial saliva (negative test) and 1 ng/mL SARS-CoV-2 spike protein (positive test) in artificial saliva after 4 h of incubation.

The PDA sensing chips in this study were divided into three separate experimental groups with different molar concentrations of PCDA, PCDA-NHS, and DMPC (Table 1). The chemicals for each experimental group were dissolved in 15 mL chloroform. The PVDF transfer membranes were cut into 1-cm by 1-cm squares and separated into three groups. Each group of membranes was dipped into the chloroform solution of one of the three experimental PCDA-NHS groups and allowed to dry at room temperature for 6 h (Figure 1a). After preparation, PDA coated on the PVDF membrane was incubated in SARS-CoV-2 spike protein antibody solution. Briefly, the stock solution containing the SARS-CoV-2 Spike S1 antibody was diluted to an antibody concentration of 10 μg/mL in PBS buffer. Each group of sensor membranes was placed in separate containers containing an equal volume of the antibody solution and was incubated overnight at 4 °C (Figure 1b). The remaining NHS sites were deactivated by incubation in a solution of 18.3 nM ethanolamine in PBS buffer for 1 h at room temperature. The membranes were then rinsed with deionized water and dried in the dark. Sensor membranes were photopolymerized by exposure to a 254 nm UV lamp for 1 min, resulting in a colorimetric shift of the membranes to a dark blue hue (Figure 1c).

### 2.5. PDA Sensor Response to the SARS-CoV-2 Spike Protein

Artificial saliva with mucin was prepared as directed by the manufacturer’s instructions. The lyophilized SARS-CoV-2 spike protein powder was reconstituted as per the manufacturer’s instructions. It was diluted to make solutions with final concentrations of 1000 ng/mL, 100 ng/mL, 10 ng/mL, and 1 ng/mL of spike protein in both artificial saliva and PBS buffer. Sensing chips from each PCDA-NHS group were submerged in 1 mL of solutions containing specific concentrations of the spike protein in artificial saliva and separately in PBS buffer. The sensing chips were in a dark, room-temperature environment for 4 h. The target spike proteins bind to the PDA and there is a steric effect at the PDA surface, resulting in changes in the PDA ene—yne backbone. The change in the structure of PDA is indicated by a color change (Figure 1d). All of the tests for each concentration and solvent type were performed in triplicate. Smartphone images of each sensing chip were obtained each hour under uniform lighting conditions.

### 2.6. Specificity of Sensor to the SARS-CoV-2 Spike Protein in the Presence of the MERS-CoV Spike Protein

In the procedure for testing sensor response to the SARS-CoV-2 spike protein, sensing chips from each PCDA-NHS group were submerged in 1 mL of solutions containing 100 ng/mL of the MERS-CoV spike protein in artificial saliva and separately in PBS buffer. The sensing chips were in a dark, room-temperature environment for the 4 h duration of testing. The tests for each solvent type were performed in triplicate. Smartphone images of each sensing chip were obtained each hour under uniform lighting conditions.

### 2.7. Effects of Temperature and pH on the Performance of the Sensor

The sensors from each group of membranes were exposed to temperatures of 30 °C, 40 °C, 50 °C, 60 °C, and 70 °C for 5 min each using a controlled hot plate. One sensing chip from each PCDA-NHS group was exposed to each temperature. Following exposure to the heating plate, smartphone images were obtained of the sensors under uniform lighting conditions.

Various solutions of known pH were prepared by dropwise addition of 0.1 M sodium hydroxide (NaOH) and 0.1 M hydrochloric acid (HCl) to 50 mL of deionized water. Real-time pH measurements of the solutions were obtained using a Vernier pH Sensor and the LabVIEW computer software. Solutions were created with a measured pH of 3, 5, 7, 9, 11, and 13. Sensors were exposed to each pH solution for 4 h, with one sensing chip from each PCDA-NHS group exposed to each pH solution. Following exposure to the pH solutions, smartphone images were obtained of the sensors under uniform lighting conditions.

### 2.8. Statistical Analyses

RGB data were extracted from the collected images using Adobe Photoshop CC 2021. In the Photoshop software, the Average Blur filter was used to determine the average RGB signal of each 1-cm by 1-cm sensing chip. The averaged RGB data for each sensor was then organized in the spreadsheet software for further statistical analysis. NMR spectra were plotted in the MestreNova software, FT-IR spectra were plotted in the OriginPro software, and all other graphs were generated in GraphPad Prism 8. One-sample *t*-tests were performed in the Minitab 20 software with a hypothesized red chromatic shift (RCS) percentage mean of 0, and an alternative hypothesis of the sample RCS means more significant than the hypothesized RCS mean. An α of 0.05 was used for all statistical tests.

## 3. Results and Discussion

### 3.1. Characterization of PCDA-NHS

**Figure 2:**
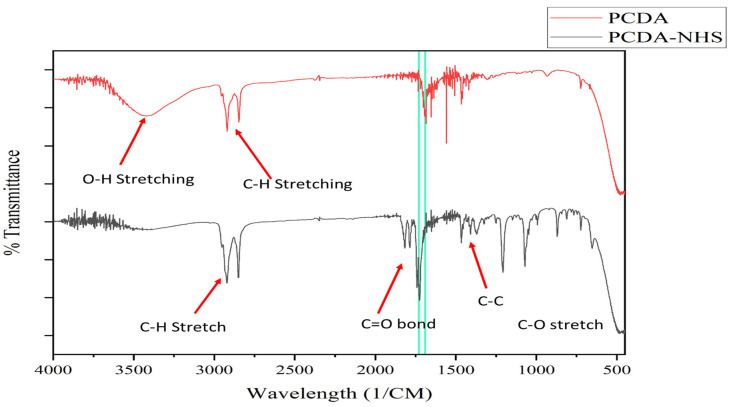
FT-IR spectra of PCDA and PCDA-NHS. FT-IR spectra were obtained from 3 mg of PCDA and 3 mg of PCDA-NHS separately dissolved in ethyl acetate. The peak in the PCDA spectra at 1690 cm^−1^ results from carboxylic acid stretching, which is not present in the spectra for PCDA-NHS. The peak in the PCDA-NHS spectra at 1729 cm^−1^ results from the NHS ester bond, which is not present in the spectra for PCDA. Two vertical lines are displayed to emphasize these peaks.

**Figure 3:**
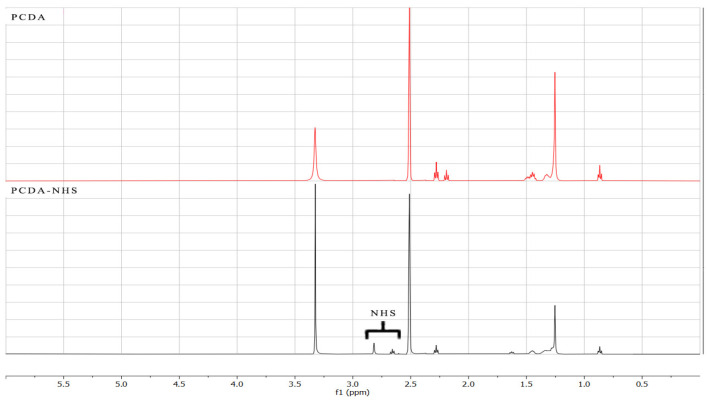
^1^H-NMR spectra of PCDA and PCDA-NHS. NMR spectra (500 MHz) were obtained from 3 mg of PCDA, and 3 mg of PCDA-NHS separately dissolved in DMSO-d_6_. The peak in the PCDA-NHS spectra at 2.8 ppm results from the NHS ester bond, which is not present in the spectra for PCDA.

The PCDA-NHS was synthesized using NHS and PCDA monomers via an esterification reaction. Noteworthily, the PCDA serves as the colorimetric sensing compound of PDA that binds to proteins. In this regard, the PCDA-NHS acts as a binding site for the SARS-CoV-2 spike protein antibody. The PCDA-NHS structure was confirmed using FT-IR and ^1^H-NMR spectra. The FT-IR spectra for the PCDA compound show unique carboxylic acid stretching (−COOH) at 1690 cm^−1^, but this carboxylic acid peak was absent in the PCDA-NHS spectra. Additionally, PCDA-NHS showed a unique peak at 1729 cm^−1^, correlating to the NHS ester bond (Figure 2). In the NMR spectra, PCDA-NHS exhibited a unique peak at 2.8 ppm, correlating to the NHS ester bond, but this peak was absent in the spectra for PCDA (Figure 3). Furthermore, thin-layer chromatography (TLC) was performed to confirm the completion of PCDA-NHS synthesis (Figure S1 in Supplementary Materials). The retardation factor (R_f_) measured for PCDA was 0.42 and the R_f_ value for PCDA-NHS was 0.30. NHS was not observed since it is not UV-active and had no measurable R_f_ value. The difference in distances traveled by the PCDA and PCDA-NHS samples indicates the greater polarity of PCDA-NHS than PCDA and thus suggests that the reaction was completed.

### 3.2. Effect of Environmental Factors on PDA Sensor

**Figure 4:**
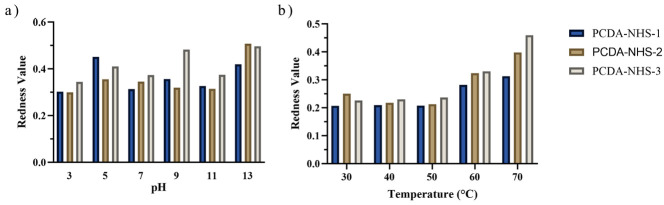
Effect of pH and temperature on redness value. Sensors from each PCDA-NHS group were exposed to solutions of various pH (**a**) and were also exposed to various temperatures (**b**).

The influence of pH and temperature on the antibody-immobilized PDA sensor was investigated to determine the optimum performance of the sensor. Figure 4a,b show the performance effect of pH and temperature within 3 to 13 and 30 °C to 70 °C, respectively. In the pH experiment, sensors exposed to solutions with pH of 3 through 7 generally showed an upward increase in the redness value from PCDA-NHS 1 to PCDA-NHS-2 and PCDA-NHS-3. This observation is attributed to the different concentrations of PCDA-NHS in each experimental group. Meanwhile, sensors exposed to solutions with pH of 7 through 13 showed pronounced redness values reaching 0.5 (Figure 4a). It is worth noting that the PDA-PVDF membrane, in its undisrupted state, exhibits blue color in acidic conditions [24]. The change from blue to red occurs in the presence of increasing pH, which perturbs the sensor to change its electronic state.

Similar trends were observed for increasing temperature. The PDA-PVDF membrane also exhibited a color transition from blue to red with increasing temperature. The increasing temperature alters the electronic state (HUMO-LUMO) of the PDA backbone, corresponding to a blue to red color change [5,24]. An increase in temperature corresponded with an increase in the redness of the PDA-PVDF membrane for temperatures from 50 °C to 70 °C, with the PCDA-NHS-3 sensor group displaying the largest redness values, followed by sensors from the PCDA-NHS-2 and PCDA-NHS-1 groups, respectively (Figure 4b). These results are attributed to the ability of the PCDA component of PDA to undergo photopolymerization [3,24]. As a result, there is a change in its chromatic properties.

As demonstrated in Figure 4, pH and temperature influence the chromatic properties of the sensors, despite being immobilized on PVDF membranes. The perturbation caused by these environmental factors induces conformational changes in the molecular structure of the polymer, such as side-chain packing and orientation, and this modified electronic state results in a chromatic transition of the sensor [24].

### 3.3. PDA Biosensor for SARS-CoV-2 Detection

**Figure 5:**
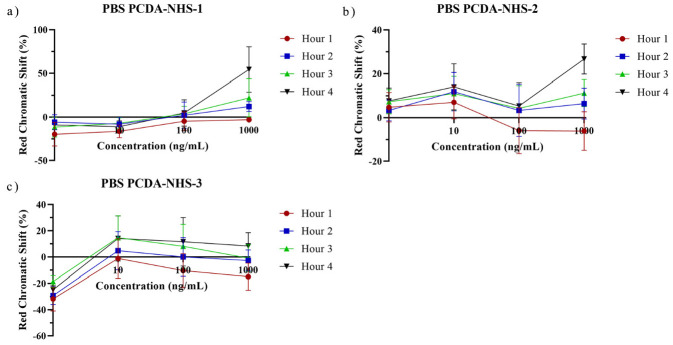
Hourly red chromatic shift for PBS samples. RCS data obtained from sensors of PCDA-NHS-1 (**a**), PCDA-NHS-2 (**b**), and PCDA-NHS-3 (**c**) groups exposed to 1, 10, 100, and 1000 ng/mL of SARS-CoV-2 spike protein in PBS buffer. As time increased, RCS tended to increase for sensors at each concentration.

**Figure 6:**
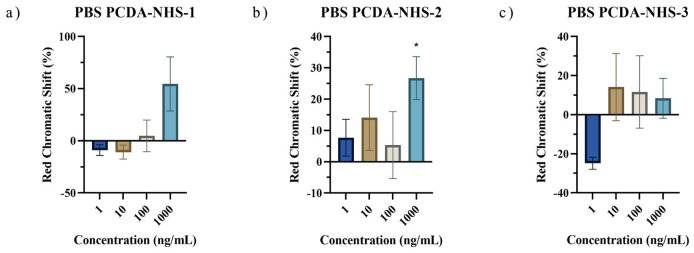
Red chromatic shift for PBS samples after 4 hours of incubation. RCS data was obtained from sensors of PCDA-NHS-1 (**a**), PCDA-NHS-2 (**b**), and PCDA-NHS-3 (**c**) groups after 4 h of incubation in solutions containing various concentrations of the SARS-CoV-2 spike protein in PBS buffer (* *p* < 0.05).

Polydiacetylene-based paper biosensors were developed to detect SARS-CoV-2 spike protein with a naked-eye colorimetric readout. It was necessary to determine the effect of PCDA-NHS concentration on the colorimetric shift of the sensors. Three different groups of sensing chips were prepared with different molar concentrations of the PCDA-NHS monomer. It was expected that the experimental group with an optimized molar ratio of PCDA-NHS (PCDA-NHS-1, PCDA-NHS-2, or PCDA-NHS-3) would produce an accurate redness shift corresponding to the efficient performance of the sensor for the detection of COVID-19 disease. The NHS ester of PCDA was synthesized to form antibody binding sites by creating reactive acylating agents, which allowed for the conjugation of the SARS-CoV-2 spike protein antibody. DMPC was incorporated into the sensor design to increase the fluidity of the membrane, with a greater fluidity allowing for enhanced sensitivity of the PDA sensor [22]. Additionally, ethanolamine was used to block non-specific absorption. The sensors were incubated in solutions containing various concentrations of the SARS-CoV-2 spike protein from 0 ng/mL to 1000 ng/mL in PBS buffer, and 1 M NaOH solution acted as the control. Images were taken of the sensors after each hour of incubation for a total of 4 h. The relative intensity of the red component (redness value), denoted as *r*, was calculated using the formula: $r=\frac{R}{R+G+B}$. Red chromatic shift (*RCS*) percentages [25] were used as quantitative parameters representing the normalized change in red chromaticity of each of the sensing pads, which allowed for the comparison of sensor results. *RCS* percentages were calculated by the following formula [26,27]: $\%\;RCS=\frac{r_{sample}-r_{negative}}{r_{positive}-r_{negative}}\times 100$. As time progressed, the *RCS* percentage tended to increase for sensors within each PCDA-NHS group at each concentration of the spike protein (Figure 5). The calculated *RCS* percentages from sensors of each PCDA-NHS group, concentration, and solution background were averaged and plotted with their corresponding standard deviation. The relatively large error bars obtained from RGB data for the PCDA-NHS-2 and PCDA-NHS-3 groups can be partially attributed to color error from smartphone imaging [28]. One-sample *t*-tests were run for RCS data obtained from the sensors of each PCDA-NHS group and concentration after 4 h of incubation (Figure 6).

### 3.4. Specificity of the PDA Sensor

**Figure 7:**
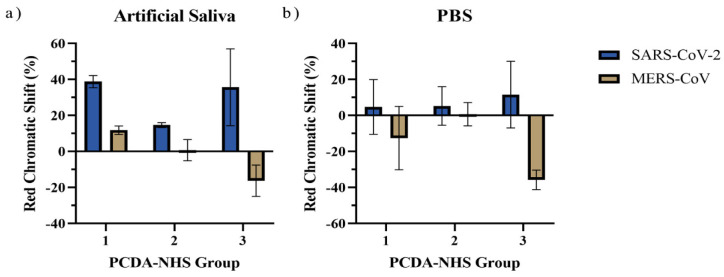
Red chromatic shift for sensors exposed to SARS-CoV-2 and MERS-CoV spike proteins. RCS data was obtained from sensors of each PCDA-NHS group after 4 h of incubation in artificial saliva (**a**) and PBS (**b**) solutions containing 100 ng/mL SARS-CoV-2 spike protein or 100 ng/mL MERS-CoV spike protein. Sensors exposed to the MERS-CoV spike protein did not produce colorimetric shifts discernible by the naked eye.

Sensors from each PCDA-NHS group were exposed to PBS solutions containing 100 ng/mL of the MERS-CoV spike protein to determine the specificity of the sensor for the SARS-CoV-2 spike protein. For control experiments, sensors from each PCDA-NHS group were exposed to PBS buffer without spike protein. All experiments were performed in triplicate. The sensors exposed to the PBS buffer had no significant color transitions without the spike protein. In addition, sensors exposed to the MERS-CoV spike protein experienced much lower RCS percentages than sensors exposed to the same concentration of the SARS-CoV-2 spike protein in artificial saliva (Figure 7). This was further supported by naked-eye observations of the colorimetric outputs of the sensors, in which sensors exposed to the MERS-CoV spike protein did not produce observable redshifts.

### 3.5. Performance of PDA Sensor for SARS-CoV-2 Detection in Artificial Saliva

**Figure 8:**
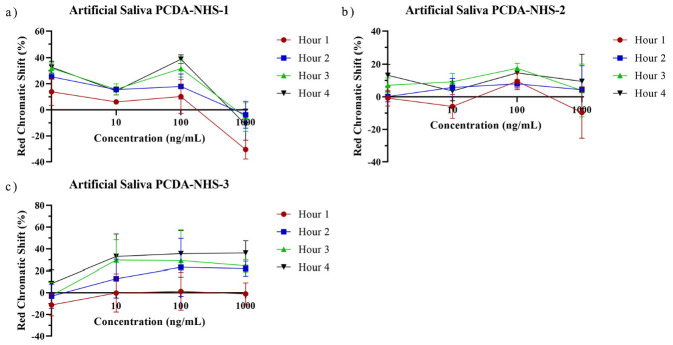
Hourly red chromatic shift for artificial saliva samples. RCS data obtained from sensors of PCDA-NHS-1 (**a**), PCDA-NHS-2 (**b**), and PCDA-NHS-3 (**c**), groups exposed to 1, 10, 100, and 1000 ng/mL of SARS-CoV-2 spike protein in artificial saliva. As time increased, RCS tended to increase for sensors at each concentration.

**Figure 9:**
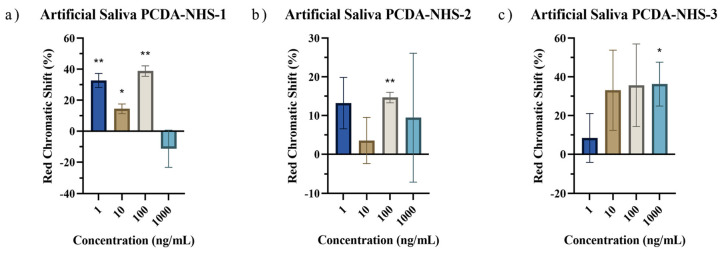
Red chromatic shift for artificial saliva samples after 4 hours of incubation. RCS data was obtained from sensors of PCDA-NHS-1 (**a**), PCDA-NHS-2 (**b**), and PCDA-NHS-3 (**c**), groups after 4 h of incubation in solutions containing various concentrations of the SARS-CoV-2 spike protein in artificial saliva (* *p* < 0.05, ** *p* < 0.01).

To investigate the real application of the PDA sensors in artificial spiked saliva, sensors from each PCDA-NHS group were exposed to solutions containing various concentrations of the SARS-CoV-2 spike protein from 0 ng/mL to 1000 ng/mL in artificial saliva. As time progressed, the RCS percentage increased for sensors within each PCDA-NHS group at each concentration of the spike protein (Figure 8). One-sample *t*-tests were run for RCS data obtained from sensors of each PCDA-NHS group and concentration after 4 h of incubation. *p*-values obtained from the *t*-tests indicate a significant shift in red chromaticity for the selected concentration gradient of the spike protein in artificial saliva (Figure 9). In the PCDA-NHS-1 group in artificial salivary background, significant results were obtained for concentrations of 1 ng/mL, 10 ng/mL, and 100 ng/mL of the spike protein.

It was determined that the optimal molar concentration of PCDA-NHS in the polydiacetylene polymer to maximize the RCS of the sensor output, while maintaining stability in the presence of varying environmental conditions, was 10% (PCDA-NHS-1 group). This is supported by previous studies that have also utilized a 10% molar concentration of modified PCDA in PDA-based biosensors [5,11,23,29]. The low *p*-values obtained by one-sample *t*-tests for the PCDA-NHS-1 sensors in artificial saliva solutions, as well as the relatively small error bars, indicate the distinguishability of the sensor output from the negative control. Furthermore, for all concentrations of the SARS-CoV-2 spike protein, except the 1000 ng/mL solution, distinct blue to red transitions in the PCDA-NHS-1 sensor outputs were visible by the naked eye after 4 h of incubation. This indicates that an RCS percentage of approximately 10% or greater is discernible by naked-eye observation, as reported in previous studies [30,31].

**Figure 10:**
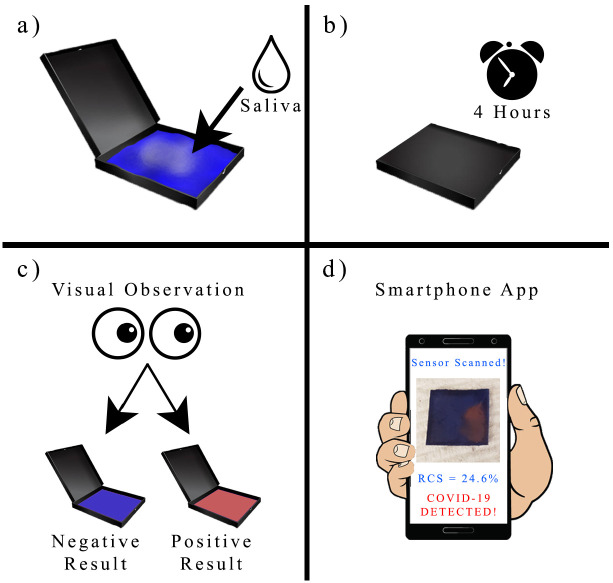
Schematic representation of the practical application of the developed biosensor. Users of this sensor would expectorate onto a PDA paper sensing pad (**a**), close the lid of the device for 4 h (**b**), and visually observe if the sensing pad changes color, with a blue pad indicating a negative result and a red pad indicating a positive result (**c**). Users can optionally use a smartphone app to scan the sensing pad and return instantaneous results (**d**).

The detection limit of the sensor was determined to be the lowest concentration of the spike protein, which resulted in a visually-discernible color transition. For the PCDA-NHS-1 group, the detection limit of the sensor by naked-eye observation after 4 h of incubation was determined to be 1 ng/mL, which is comparable to the detection limits obtained by other COVID-19 biosensors (Table 2). It was concluded that 1000 ng/mL of spike protein was too concentrated to be accurately and precisely detected by the sensors. Thus, the detection range of this sensor was determined to be 1 ng/mL to 100 ng/mL, which satisfies the diagnostic requirements of POC testing for COVID-19 [32]. The significance of this finding indicates the potential use of this biosensor in homes without access to advanced medical equipment (Figure 10). Furthermore, this finding is also significant because it suggests the potential use of this biosensor in developing regions of the world without access to consumer electronics, such as smartphones. Although smartphone imaging could be used to assess smaller levels of RCS, unaided visual observations of the sensor after 4 h of incubation can indicate whether SARS-CoV-2 spike protein is present in the saliva sample with a detection range of 1 to 100 ng/mL.

This study utilized artificial saliva for diagnostic testing by spiking the fluid with various concentrations of the SARS-CoV-2 spike protein. The enzymes present in human saliva can interfere with the detection of COVID-19. Further research could reveal the effect of salivary enzymes on the PDA sensor at relevant concentrations of the SARS-CoV-2 virus.

The PDA sensor design can also be applied for the detection of Alphacoronaviruses (α-CoV), Betacoronaviruses (β-CoV), Gammacoronaviruses (γ-CoV), and Deltacoronaviruses (δ-CoV) [38,39,40]. The detection capabilities of this sensor may be improved with the creation of a smartphone application capable of instantaneous RCS readout. This smartphone application could provide a user-friendly interface to take images of the sensor after exposure to saliva. Using computer algorithms could allow smartphone applications to mitigate the effects of unequal lighting by preventing the user from taking a photo in improper lighting conditions and performing minor lighting adjustments to match its preset brightness levels. In addition, the application could calculate the RCS percentage within seconds using pre-programmed negative and positive control values. Thus, this smartphone application could facilitate in-field analysis and at-home analysis by providing more accurate measurements of the sensor’s colorimetric output.

## 4. Conclusions

This study demonstrated a paper-based polydiacetylene biosensor for detecting the SARS-CoV-2 spike protein in saliva. The polydiacetylene-based sensors exhibited visibly discernible blue to red color transitions in the presence of various concentrations of the SARS-CoV-2 spike protein. The molar concentration of PCDA-NHS in the polymer was optimized (10%), and the detection range of the biosensor was 1 to 100 ng/mL with naked-eye observations. The results from the PDA-coated PVDF membranes exhibit varying degrees of color changes, depending on the concentration of the SARS-CoV-2 spike protein and the detection time. Smartphone images of the PDA-coated PVDF membranes were evaluated using a computer program to obtain accurate quantitative data. The experimental results showed high specificity of the fabricated PDA sensors, which were minimally influenced by pH and temperature. The results of this study could be applied in developing countries due to the low-cost, high sensitivity, and user-friendly design of this rapid, colorimetric sensing device.

## Figures and Tables

**Table 1:** Molar compositions of PCDA-NHS groups. Each PCDA-NHS group consists of different molar ratios of PCDA, PCDA-NHS, and DMPC. The PCDA-NHS-1 group has a 10% molar concentration of PCDA-NHS, whereas the PCDA-NHS-2 and PCDA-NHS-3 groups have a 20% and 30% molar concentration of PCDA-NHS, respectively.

	PCDA	PCDA-NHS	DMPC
PCDA-NHS-1 Group	561.9 mg (1.5 mmol)	146.9 mg (0.3 mmol)	813.5 mg (1.2 mmol)
PCDA-NHS-2 Group	449.5 mg (1.2 mmol)	293.8 mg (0.6 mmol)	813.5 mg (1.2 mmol)
PCDA-NHS-3 Group	337.1 mg (0.9 mmol)	440.7 mg (0.9 mmol)	813.5 mg (1.2 mmol)

**Table 2:** Comparison of active biosensors for SARS-CoV-2 detection. The sensor design, detection target, sample type, and detection limit are presented for various biosensors designed to detect SARS-CoV-2 in patients.

Sensor Design	Detection Target	Sample Type	Detection Limit	Reference
Magnetic beads-based biosensor	Spike (S) protein and nucleocapsid (N) protein	Saliva	19 ng/mL for S protein, 8 ng/mL for N protein	Fabiani et al. [33]
Field-effect transistor	Spike protein	Nasal samples	100 fg/mL	Seo et al. [34]
Colorimetric bioassay utilizing surface plasmon resonance	Nucleic acid	Isolated RNA	180 ng/mL	Moitra et al. [35]
Paper-based electrochemical biosensor	Antibody	Human serum	1 ng/mL	Yakoh et al. [36]
Optofluidic fluorescence biosensor	Antibody	Human serum	12.5 ng/mL	Song et al. [37]
Polydiacetylene-based paper biosensor	Spike protein	Saliva	1 ng/mL	This study

## Data Availability

The data presented in this study are available on request from the corresponding author.

## Supplementary Materials

The following supporting information can be downloaded at: https://www.mdpi.com/article/10.3390/bios12100804/s1, Figure S1. Thin-layer chromatography of PCDA, NHS, and PCDA-NHS. The distances traveled by the PCDA and PCDA-NHS samples are indicated by horizontal lines. The NHS sample was not shown to be UV-active. PCDA exhibited an R_f_ value of 0.42 and PCDA-NHS exhibited an R_f_ value of 0.30. The different distances traveled by PCDA and PCDA-NHS result from their different polarities and indicate that the reaction was completed.
