# Supplementary material for: Colorimetric Detection of the SARS-CoV-2 Virus (COVID-19) in Artificial Saliva Using Polydiacetylene Paper Strips
Source: Biosensors (Basel). 2022 Sep 29;12(10):804. doi: 10.3390/bios12100804 (PMC9599072; doi:10.3390/bios12100804)
Supplement: Supplementary file 1 [file biosensors-12-00804-s001.zip › biosensors-1920535-supplementary.pdf]

biosensors

MDPI

Supplementary Materials

Colorimetric Detection of the SARS-CoV-2 Virus (COVID-19) in Artificial Saliva Using Polydiacetylene Paper Strips

Christopher D. Prainito [1], Gaddi Eshun [2], Francis J. Osonga [2], Daniel Isika [2], Cynthia Centeno [2], and Omowunmi A. Sadik [2,*]

[1] Current address: Harvard University, 29 Oxford Street, Cambridge, MA 02138, USA
[2] Chemistry and Environmental Science Department, New Jersey Institute of Technology, University Heights, Newark, NJ 07102, USA
* Correspondence: sadik@njit.edu

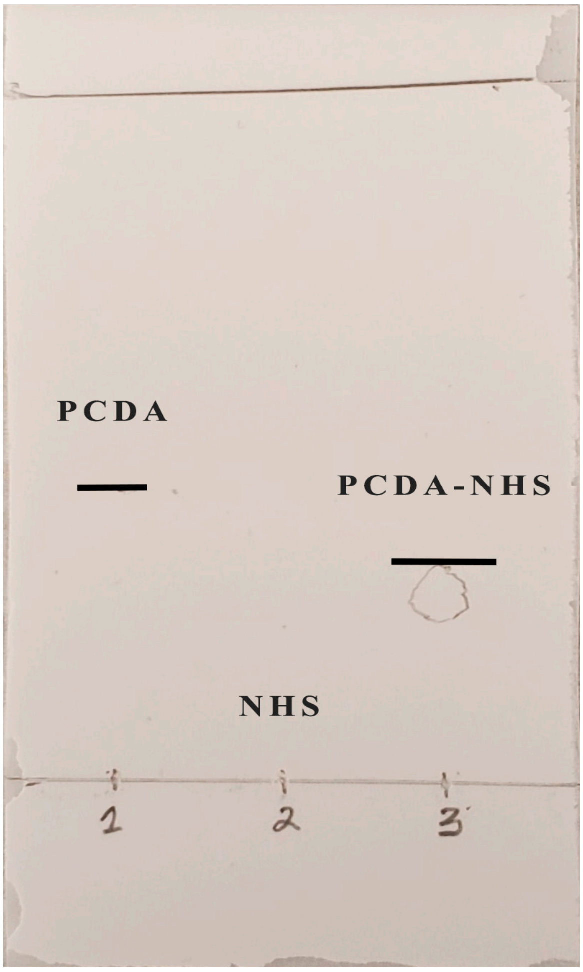

Figure S1. Thin-Layer Chromatography of PCDA, NHS, and PCDA-NHS. The distances traveled by the PCDA and PCDA-NHS samples are indicated by horizontal lines. The NHS sample was not shown to be UV-active. PCDA exhibited an R_f value of 0.42 and PCDA-NHS exhibited an R_f value of 0.30. The different distances traveled by PCDA and PCDA-NHS result from their different polarities and indicate that the reaction was completed.
